# HPLC fucoxanthin profiles of a microalga, a macroalga and a pure fucoxanthin standard

**DOI:** 10.1016/j.dib.2016.12.047

**Published:** 2016-12-29

**Authors:** Su Chern Foo, Fatimah Md. Yusoff, Maznah Ismail, Mahiran Basri, Sook Kun Yau, Nicholas M.H. Khong, Kim Wei Chan, Mahdi Ebrahimi

**Affiliations:** a Institute of Bioscience, Universiti Putra Malaysia, 43400 Serdang, Selangor Darul Ehsan, Malaysia; b Department of Aquaculture, Faculty of Agriculture, Universiti Putra Malaysia, 43400 Serdang, Selangor Darul Ehsan, Malaysia; c Department of Chemistry, Faculty of Science, Universiti Putra Malaysia, 43400 Serdang, Selangor Darul Ehsan, Malaysia; d Department of Veterinary Preclinical Sciences, Universiti Putra Malaysia, 43400 Serdang, Selangor Darul Ehsan, Malaysia

**Keywords:** High performance liquid chromatograph (HPLC), Carotenoids, Fucoxanthin, Microalgae, Macroalgae

## Abstract

Data in this article illustrate representative fucoxanthin chromatograms of a microalga, *Chaetoceros calcitrans*; a macroalga, *Saccharina japonica* and; a pure fucoxanthin standard. High performance liquid chromatography (HPLC) eluted fucoxanthin at the 7.008±0.024th min. This data article refers to the research article ‘‘Antioxidant capacities of fucoxanthin-producing algae as influenced by their carotenoid and phenolic contents’’ Foo et al. [1]; where a more comprehensive data interpretation and analysis is explained.

**Specifications Table**

**Table:** 

Subject area	Biology, Analytical Chemistry
More specific subject area	Algae carotenoids
Type of data	Figure
How data was acquired	HPLC instrument Agilent 1300 series DAD 1400 diode array detector; Agilent G1301A autosampler (Agilent Technologies Inc., GA, USA); Merck Chromolith^®^ RP-18e (3 mm × 4.6 mm i.d. 2 μm pore size).
Data format	Raw and analyzed
Experimental factors	These are described in the text description of the data
Experimental features	These are described in the text description of the data
Data source location	Laboratory of Molecular Medicine, Universiti Putra Malaysia, Serdang, 43400, Selangor, Malaysia
Data accessibility	Data with article

**Value of the data**

• Data show the HPLC separation of a characteristic and major carotenoid, fucoxanthin from a diatom (*i.e. Chaetoceros calcitrans*) and a brown seaweed (*i.e. Saccharina japonica*).
• The time of fucoxanthin elution for both algae; with reference to a pure standard is illustrated with a distinct peak.
• These data are useful for comparison with other fucoxanthin-producing species *i.e.* estimation of fucoxanthin content.
• Provides a valuable carotenoid reference for future taxonomic identification in algae.

## 1 Data

**Fig. 1:**
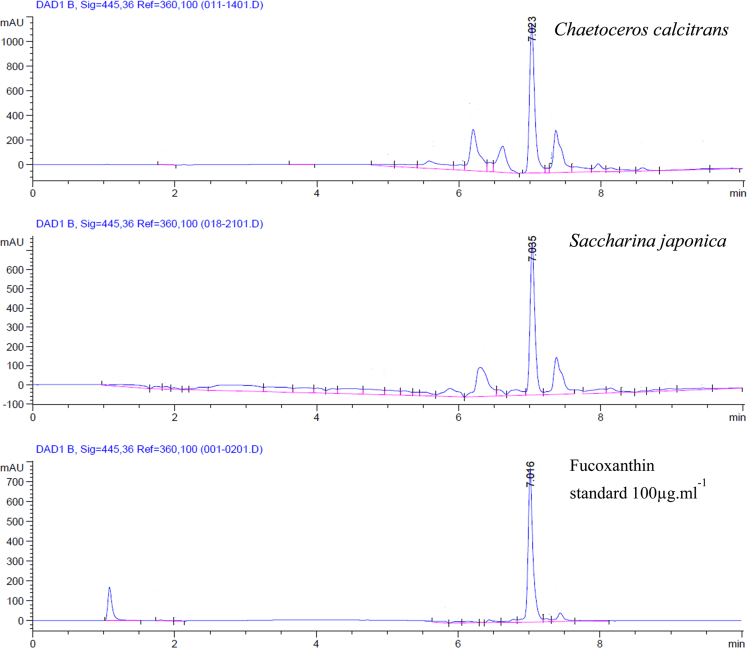
Representative fucoxanthin chromatograms showing a microalga, *Chaetoceros calcitrans* to have a significantly higher absorbance than a macroalga, *Saccharina japonica* at the 7.008±0.024th min.

Antioxidant activities of six species of fucoxanthin-producing algae were evaluated using *in vitro* antioxidant assays. Fucoxanthin concentrations from each species were quantified and analyzed using HPLC. The method described is able to elute fucoxanthin in only 15 minutes with a clear and distinctive peak (Fig. 1).

## 2 Experimental design, materials and methods

### 2.1 Sample preparation

Extracts were obtained by methanolic extraction of 1.0 g of lyophilised algae biomass respectively; as described by Foo et al. [2].

### 2.2 HPLC analysis

Samples were prepared by fully solubilizing 10 mg of dried extracts in 1 ml of methanol. Each sample was run in triplicates on a HPLC (Agilent 1300 HPLC series, Agilent Technologies Inc., GA, USA). Twenty microliters of sample was injected using an autosampler (Agilent 1300 series G1329-90010) onto a Chromolith^®^ RP-18e (3 mm × 4.6 mm i.d. 2 μm pore size) reverse phase column (Merck Millipore, KGaA Darmstadt, Germany). Carotenoids were chromatographically separated by an increasing methanol gradient, at a flow rate of 1 ml.min^-1^. The mobile phase gradient selected was 100% water (A) and 100% methanol (B): starting from 0% to 100% A in 2 min, 100% to 50% A in 3 min, 50% to 25% A in 4 min, 25% to 10% A in 6 min, 10% to 5% A in 8 min, and 0% to 100% B in 15 min. The absorbance at 445 nm for each run was recorded. The standard curve and retention times were calibrated using pure fucoxanthin standard (Sigma-Aldrich Co., St. Louis, MO, USA) solubilized in methanol at six concentrations.

### 2.3 Data processing

The automated integration software (Agilent ChemStation software, Waldbronn, Germany) was used to acquire the area under the curve (mAU*s). Results were then expressed as milligram fucoxanthin per gram dry weight biomass (mg FX.g^−1^.DW) as reported in Foo et al. [1].
