# Supplementary material for: HPLC fucoxanthin profiles of a microalga, a macroalga and a pure fucoxanthin standard
Source: Data Brief. 2016 Dec 29;10:583–6. doi: 10.1016/j.dib.2016.12.047 (PMC5219594; doi:10.1016/j.dib.2016.12.047)
Supplement: Supplementary file 1 — Supplementary material [file mmc1.pdf]

UPM
UNIVERSITI PUTRA MALAYSIA
BERILMU BERBAKTI

INSTITUT BIOSAINS
INSTITUTE OF BIOSCIENCE

17th December 2016

**Conflict of Interest Statement**

Title of paper: HPLC fucoxanthin profiles of a microalga, a macroalga and a pure fucoxanthin standard

We wish to draw the attention of the Editor to the following facts which may be considered as potential conflicts of interest and to significant financial contributions to this work. [OR] We wish to confirm that there are no known conflicts of interest associated with this publication.

We confirm that the manuscript has been read and approved by all named authors and that there are no other persons who satisfied the criteria for authorship but are not listed. We further confirm that the order of authors listed in the manuscript has been approved by all of us.

We confirm that we have given due consideration to the protection of intellectual property associated with this work and that there are no impediments to publication, including the timing of publication, with respect to intellectual property. In so doing we confirm that we have followed the regulations of our institutions concerning intellectual property.

We understand that the Corresponding Author is the sole contact for the Editorial process (including Editorial Manager and direct communications with the office). He/she is responsible for communicating with the other authors about progress, submissions of revisions and final approval of proofs. We confirm that we have provided a current, correct email address which is accessible by the Corresponding Author.

Thank you.
Sincerely,

______________________________

Prof. Dr. Fatimah Md. Yusoff, PhD
Laboratory of Marine Biotechnology
Institute of Bioscience,
Universiti Putra Malaysia
43400 UPM Serdang, Selangor, Malaysia

Institut Biosains, Universiti Putra Malaysia, 43400 UPM Serdang, Selangor Darul Ehsan, Malaysia
603-8947 2111 603-8947 2101 http://www.ibs.upm.edu.my
